# Supplementary material for: Chromothripsis during telomere crisis is independent of NHEJ, and consistent with a replicative origin
Source: Genome Res. 2019 May;29(5):737–49. doi: 10.1101/gr.240705.118 (PMC6499312; doi:10.1101/gr.240705.118)
Supplement: Supplemental Material [file supp_gr.240705.118_Supplemental_file_1.zip › contigs/annotated_contigs/DB37/contig.2.DB37_length_849_mean_cov_32.6902237927.docx]

**DB37_length_849_mean_cov_32.6902237927**

```
GAAGGCAGACCGAAGGGAAGGAGGGCATCCTAGGTGACATTTCCATACCCACGCACCCTTTACAATGCTGGGGCTGCCAGTCCACCCTG
 >chrUn_gl000220:122141-122931 - E=0e+00 p=0e+00
TACGTCAACCCACCCCCAAGAACAGCACGGTCCGGGGTGGTCCAGTCTGATCCCAACCGGCCCACCCGGGGCATCCGGTGGAAGTCTTC

GCCGGAGGATCCGAAGGCAGCATCAACGCGGTTCCCCTGGGGTCGCCCGGCAAAGGCCAGCCGGGGGAGGGTAGCGGGACGTGACGGGG

GGGTGGGGGTCGCATCCGCCTCAGAGCTCCCTGGAAGGTGGCAGGTAGCCGGTGGGGCACGCCGAGCCAGAGACGTCCGGCAGGATATA

GATCTGGAAGGCGTGTCAGTCCTCTCCCATACCTCTCCTATGGAAAATGCCAGGGCGGCGGTGGGAGCCTCGGCTGGGGGAGAAGCGGG

GACAAGGGGGAGAGGGAAGGAGGCCCTCGGGAGGTTTCGGCACCGAAAACCCACTCAGCCAAGCTCCCTCCGTGTTTCCGGGTCCAAGG

TACACCCCGGGAGACGGCAAGAGAAACGTTCACACCGTGCTTTCCGTCTTCGTGTTTATTTCTTTCATCTTTTCCATTTTACGAGAGAT

GCTCATTTCAACAACCAGACGGCGGATGTGACGGGAGAAGCGTCAAGGCCAGGAGTTTGAGACCAGCGTGAGCAACAGAGCAACACACG

TAGGAGAGCCCAGCTGAAAGAAATGAAAGAGGAGGAGGAGGAGGAGGAGGAGGAGGAGGAGGAGGAGGAGGAGGAGGA|GGAGGAGGAG

GAGGAGGAGGAGGAGGAGGAGGAGGAGAAGGAAGAAGAAGAAAAAGAA|A >chr17:67944528-67944576 + E=5e-14
```
